# Supplementary material for: Identifying protein subcellular localisation in scientific literature using bidirectional deep recurrent neural network
Source: Sci Rep. 2021 Jan 18;11:1696. doi: 10.1038/s41598-020-80441-8 (PMC7813825; doi:10.1038/s41598-020-80441-8)
Supplement: Supplementary file 1 — Supplementary Information. [file 41598_2020_80441_MOESM1_ESM.pdf]

Supplemental figures and tables

Identifying protein subcellular localisation in scientific literature using bidirectional deep recurrent neural network

Rakesh David[1,*], Rhys-Joshua D. Menezes[2], Jan De Klerk[2], Ian R. Castleden[3], Cornelia M. Hooper[3], Gustavo Carneiro[2], Matthew Gilliham[1]

[1]School of Agriculture, Food and Wine, The Waite Research Institute, ARC Centre of Excellence in Plant Energy Biology, Waite Campus, The University of Adelaide, South Australia, Australia

[2]School of Computer Science, Australian Institute for Machine Learning, The University of Adelaide, South Australia, Australia

[3]ARC Centre of Excellence in Plant Energy Biology, The University of Western Australia, WA, Australia

*Corresponding author: rakesh.david@adelaide.edu.au

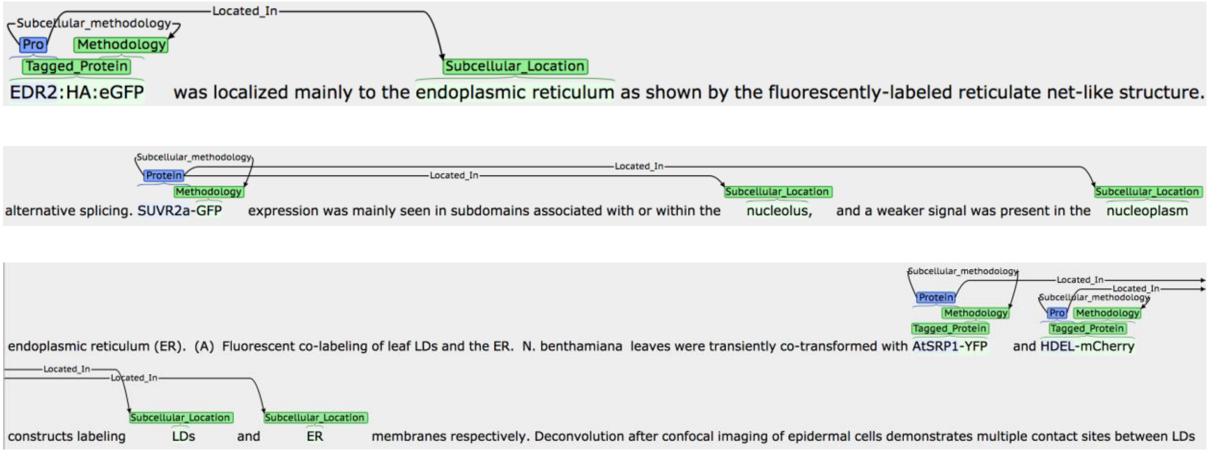

Supplemental Figure 1: Examples of BRAT annotation using extracted text from the SUBA corpus. (A) Triplet entity group representing a tagged protein (concatenation of protein and methodology) localised to a single subcellular component. (B) Triplet entity group containing a tagged protein and two subcellular components. (C) Two triplet groups containing two tagged proteins localised to distinct subcellular compartments.

Table S1: Names derived from the GO subcellular components annotations used in the form of regular expression terms to identify subcellular locations together with co-occurring proteins and experimental methodologies in the SUBA and CropPAL corpus.

**Major compartments**	**Child location**	**Grandchild locations**
cytoskeleton (GO:0005856)	intermediate filament (GO:0005882) microtubules (GO:0005884) actin filament (GO:0005884)	
cytosol (GO:0005829)	cell plate (GO:0009504) cytosolic ribosomes (GO:0022626)	
endoplasmic reticulum (GO:0005783)	ER lumen (GO:0005788) ER membrane (GO:0005789)	
extracellular (GO:0005576)	apoplast (GO:0048046) cell wall (GO:0009505)	
Golgi	Golgi apparatus (GO:0005794) trans-Golgi network (GO:0005802)	Golgi lumen (GO:0005796) Golgi membrane (GO:0000139) multivesicular body (GO:0005771) early endosome (GO:0005769)
mitochondrion (GO:0005739)	mitochondrial envelope (GO:0005740)	mitochondrial inner membrane (GO:0005743) mitochondrial outer membrane (GO:0005741)
	mitochondrial matrix (GO:0005759)	
nucleus (GO:0005634)	nuclear envelope (GO:0005635)	nuclear inner membrane (GO:0005637) nuclear outer membrane (GO:0005640)
	nuclear matrix (GO:0016363)	chromatin (GO:0000785) nucleolus (GO:0005730)
peroxisome (GO:0005777)	peroxisomal membrane	

	(GO:0005778)	
	peroxisome matrix (GO:0005782)	
plasma membrane (GO:0005886)		
plastid (GO:0009536)	plastid envelope (GO:0009526) plastid stroma (GO:0009532) plastid thylakoid (GO:0031976)	plastid inner membrane (GO:0009528) plastid outer membrane (GO:0009527) plastoglobules (GO:0010287) plastid thylakoid lumen (GO:0031978) plastid thylakoid membrane (GO:0055035)
vacuole (GO:0000325)	vacuole membrane (GO:0009705) vacuole lumen (GO:0000330)	

Table S2: Regular expression terms to identify experimental methodologies for protein subcellular location data from the SUBA and CropPAL corpus.

**Experimental Methodology**	**Regular expression query terms**
GFP	“Green fluorescent protein” OR GFP OR FP OR fluorescence OR “fluorescent protein”
Mass-spectrometry	MS/MS OR MS OR spectrometry OR “mass spectrometry”

Table S3: Bidirectional LSTM scores for SUBA and CropPAL datasets

	SUBA				CropPAL			
	**Accuracy**	**Precision**	**Recall**	**F1**	**Accuracy**	**Precision**	**Recall**	**F1**
Run1	0.9007	0.9407	0.8523	0.8944	0.9362	0.9259	0.9615	0.9434
Run2	0.7914	0.9479	0.6107	0.7429	0.7872	0.9444	0.6538	0.7727
Run3	0.8377	0.8906	0.7651	0.8231	0.8936	0.8889	0.9231	0.9057
Run4	0.8709	0.9661	0.7651	0.8539	0.8936	0.8889	0.9231	0.9057
Run5	0.8709	0.9825	0.7517	0.8517	0.9362	0.8966	1.0000	0.9455
Run6	0.8742	0.8881	0.8523	0.8699	0.8723	0.8571	0.9231	0.8889
Run7	0.8742	0.9826	0.7584	0.8561	0.8936	0.8889	0.9231	0.9057
Run8	0.8742	0.9826	0.7584	0.8561	0.8511	0.8519	0.8846	0.8679
Run9	0.8808	0.9520	0.7987	0.8686	0.8723	0.8846	0.8846	0.8846
Run10	0.8841	0.9831	0.7785	0.8689	0.8936	0.8889	0.9231	0.9057
Run11	0.8874	0.9197	0.8456	0.8811	0.9362	0.8966	1.0000	0.9455
Run12	0.8874	0.9389	0.8255	0.8786	0.9149	0.8929	0.9615	0.9259
Run13	0.8907	0.9462	0.8255	0.8817	0.9149	0.8929	0.9615	0.9259
Run14	0.8907	0.9531	0.8188	0.8809	0.9362	0.9259	0.9615	0.9434
Run15	0.8940	0.9209	0.8591	0.8889	0.8723	0.8846	0.8846	0.8846
Run16	0.8940	0.9916	0.7919	0.8806	0.8511	0.8800	0.8462	0.8627
Run17	0.9007	0.9760	0.8188	0.8905	0.8723	0.9167	0.8462	0.8800
Run18	0.9040	0.9348	0.8658	0.8990	0.8723	0.8333	0.9615	0.8929
Run19	0.9040	0.9688	0.8322	0.8953	0.8723	0.8571	0.9231	0.8889
Run20	0.9106	0.9552	0.8591	0.9046	0.8511	0.8800	0.8462	0.8627
Run21	0.9106	0.9621	0.8523	0.9039	0.8723	0.8571	0.9231	0.8889
Run22	0.9139	0.9424	0.8792	0.9097	0.9574	0.9286	1.0000	0.9630
Run23	0.9172	0.9429	0.8859	0.9135	0.9362	0.9259	0.9615	0.9434
Run24	0.9172	0.9493	0.8792	0.9129	0.9362	0.9259	0.9615	0.9434
Run25	0.9172	0.9559	0.8725	0.9123	0.9362	0.9259	0.9615	0.9434
Run26	0.9205	0.9562	0.8792	0.9161	0.9149	0.8929	0.9615	0.9259
Run27	0.9205	0.9562	0.8792	0.9161	0.8936	0.8889	0.9231	0.9057
Run28	0.9205	0.9630	0.8725	0.9155	0.9362	0.9259	0.9615	0.9434
Run29	0.9272	0.9441	0.9060	0.9247	0.8936	0.8889	0.9231	0.9057
Run30	0.9305	0.9571	0.8993	0.9273	0.9362	0.8966	1.0000	0.9455
**Average**	**0.8939**	**0.9517**	**0.8280**	**0.8840**	**0.8979**	**0.8944**	**0.9256**	**0.9082**
Stdev	0.0286	0.0245	0.0615	0.0367	0.0376	0.0267	0.0677	0.0385

Table S4: Unidirectional LSTM scores for SUBA and CropPAL datasets

	SUBA				CropPAL			
	**Accuracy**	**Precision**	**Recall**	**F1**	**Accuracy**	**Precision**	**Recall**	**F1**
Run1	0.8046	0.9500	0.6376	0.7631	0.9149	0.9231	0.9231	0.9231
Run2	0.8212	0.9612	0.6644	0.7857	0.8511	0.8519	0.8846	0.8679
Run3	0.8377	0.9237	0.7315	0.8165	0.8723	0.8846	0.8846	0.8846
Run4	0.8377	0.9808	0.6846	0.8063	0.8298	0.8750	0.8077	0.8400
Run5	0.8543	0.9730	0.7248	0.8308	0.8723	0.9167	0.8462	0.8800
Run6	0.8543	0.9817	0.7181	0.8295	0.8511	0.8800	0.8462	0.8627
Run7	0.8576	0.9492	0.7517	0.8390	0.8723	0.8846	0.8846	0.8846
Run8	0.8576	0.9732	0.7315	0.8352	0.9149	0.8929	0.9615	0.9259
Run9	0.8609	0.9421	0.7651	0.8444	0.8936	0.8889	0.9231	0.9057
Run10	0.8642	0.9355	0.7785	0.8498	0.9149	0.8667	1.0000	0.9286
Run11	0.8642	0.9576	0.7584	0.8464	0.8723	0.8846	0.8846	0.8846
Run12	0.8675	0.9739	0.7517	0.8485	0.8085	0.8696	0.7692	0.8163
Run13	0.8709	0.9583	0.7718	0.8550	0.8936	0.8889	0.9231	0.9057
Run14	0.8709	0.9661	0.7651	0.8539	0.8723	0.8846	0.8846	0.8846
Run15	0.8742	0.9440	0.7919	0.8613	0.8511	0.8800	0.8462	0.8627
Run16	0.8742	0.9587	0.7785	0.8593	0.9149	0.9231	0.9231	0.9231
Run17	0.8808	0.9520	0.7987	0.8686	0.8298	0.8750	0.8077	0.8400
Run18	0.8874	0.9389	0.8255	0.8786	0.9362	0.9259	0.9615	0.9434
Run19	0.8940	0.9535	0.8255	0.8849	0.9149	0.8929	0.9615	0.9259
Run20	0.8940	0.9680	0.8121	0.8832	0.8936	0.8889	0.9231	0.9057
Run21	0.8974	0.9155	0.8725	0.8935	0.8511	0.8800	0.8462	0.8627
Run22	0.9007	0.9343	0.8591	0.8951	0.9149	0.8667	1.0000	0.9286
Run23	0.9007	0.9542	0.8389	0.8929	0.8723	0.9167	0.8462	0.8800
Run24	0.9073	0.9007	0.9128	0.9067	0.9149	0.9231	0.9231	0.9231
Run25	0.9106	0.9552	0.8591	0.9046	0.8723	0.8846	0.8846	0.8846
Run26	0.9106	0.9766	0.8389	0.9025	0.9362	0.9259	0.9615	0.9434
Run27	0.9172	0.9429	0.8859	0.9135	0.8723	0.8846	0.8846	0.8846
Run28	0.9172	0.9697	0.8591	0.9110	0.8723	0.8846	0.8846	0.8846
Run29	0.9205	0.9562	0.8792	0.9161	0.9149	0.9231	0.9231	0.9231
Run30	0.9305	0.9507	0.9060	0.9278	0.9149	0.8929	0.9615	0.9259
**Average**	**0.8780**	**0.9532**	**0.7926**	**0.8635**	**0.8837**	**0.8920**	**0.8987**	**0.8953**
Stdev	0.0307	0.0190	0.0711	0.0403	0.0329	0.0205	0.0567	0.0325
